# New examples of ferroelectric nematic materials showing evidence for the antiferroelectric smectic-Z phase

**DOI:** 10.1038/s41598-024-54832-0

**Published:** 2024-02-23

**Authors:** Pierre Nacke, Atsutaka Manabe, Melanie Klasen-Memmer, Xi Chen, Vikina Martinez, Guillaume Freychet, Mikhail Zhernenkov, Joseph E. Maclennan, Noel A. Clark, Matthias Bremer, Frank Giesselmann

**Affiliations:** 1 Institute of Physical Chemistry, University of Stuttgart, Pfaffenwaldring 55, 70569 Stuttgart, Germany; 2 Display Solutions, Merck Electronics KGaA, 64293 Darmstadt, Germany; 3 Individual researcher (Since 01.01.22), 64625 Bensheim, Germany; 4 Department of Physics and Soft Materials Research Center, University of Colorado, Boulder, CO 80309 USA; 5 Brookhaven National Laboratory, National Synchrotron Light Source-II, Upton, NY 11973 USA

**Keywords:** Soft materials, Liquid crystals, Structure of solids and liquids, Ferroelectrics and multiferroics

## Abstract

We present a new ferroelectric nematic material, 4-((4′-((trans)-5-ethyloxan-2-yl)-2′,3,5,6′-tetrafluoro-[1,1′-biphenyl]-4-yl)difluoromethoxy)-2,6-difluorobenzonitrile (AUUQU-2-N) and its higher homologues, the molecular structures of which include fluorinated building blocks, an oxane ring, and a terminal cyano group, all contributing to a large molecular dipole moment of about 12.5 D. We observed that AUUQU-2-N has three distinct liquid crystal phases, two of which were found to be polar phases with a spontaneous electric polarization **P**_**s**_ of up to 6 µC cm^–2^. The highest temperature phase is a common enantiotropic nematic (N) exhibiting only field-induced polarization. The lowest-temperature, monotropic phase proved to be a new example of the ferroelectric nematic phase (N_F_), evidenced by a single-peak polarization reversal current response, a giant imaginary dielectric permittivity on the order of 10^3^, and the absence of any smectic layer X-ray diffraction peaks. The ordinary nematic phase N and the ferroelectric nematic phase N_F_ are separated by an antiferroelectric liquid crystal phase which has low permittivity and a polarization reversal current exhibiting a characteristic double-peak response. In the polarizing light microscope, this antiferroelectric phase shows characteristic zig-zag defects, evidence of a layered structure. These observations suggest that this is another example of the recently discovered smectic Z_A_ (SmZ_A_) phase, having smectic layers with the molecular director parallel to the layer planes. The diffraction peaks from the smectic layering have not been observed to date but detailed 2D X-ray studies indicate the presence of additional short-range structures including smectic C-type correlations in all three phases—N, SmZ_A_ and N_F_—which may shed new light on the understanding of polar and antipolar order in these phases.

## Introduction

In his 1916 attempt on a theory of nematic liquid crystals^1^, Born assumed that interactions between the permanent longitudinal dipoles of rod-shaped molecules were the driving force for the spontaneous orientational ordering of the molecular long axes along the nematic director **n**. In this picture, the longitudinal dipole moments add up to a net spontaneous electric polarization **P**_**s**_ along **n**, such that opposite directions **+ n** and **– n** can now be distinguished by the direction of **P**_**s**_. The presence of a spontaneous polarization, whose direction can be reversed by the action of an electric field, makes this nematic a ferroelectric nematic phase.

The predictions of Born's theory were, however, contradicted by several experimental findings, including the observation of half-integer disclinations in common nematics^2^, showing that sign reversal of the director, **+ n** → **– n**, is a symmetry operation of the phase. The directions of **+ n** and **– n** are thus physically indistinguishable (sign invariance of **n**) and the long-range orientational order of common nematics is purely axial rather than polar. In the 1960s, the Maier-Saupe theory^3^ provided a mean-field description of (non-polar) long-range order in nematic liquid crystals and Born's theory seemed destined to remain nothing more than an historical footnote.

**Figure 1:**
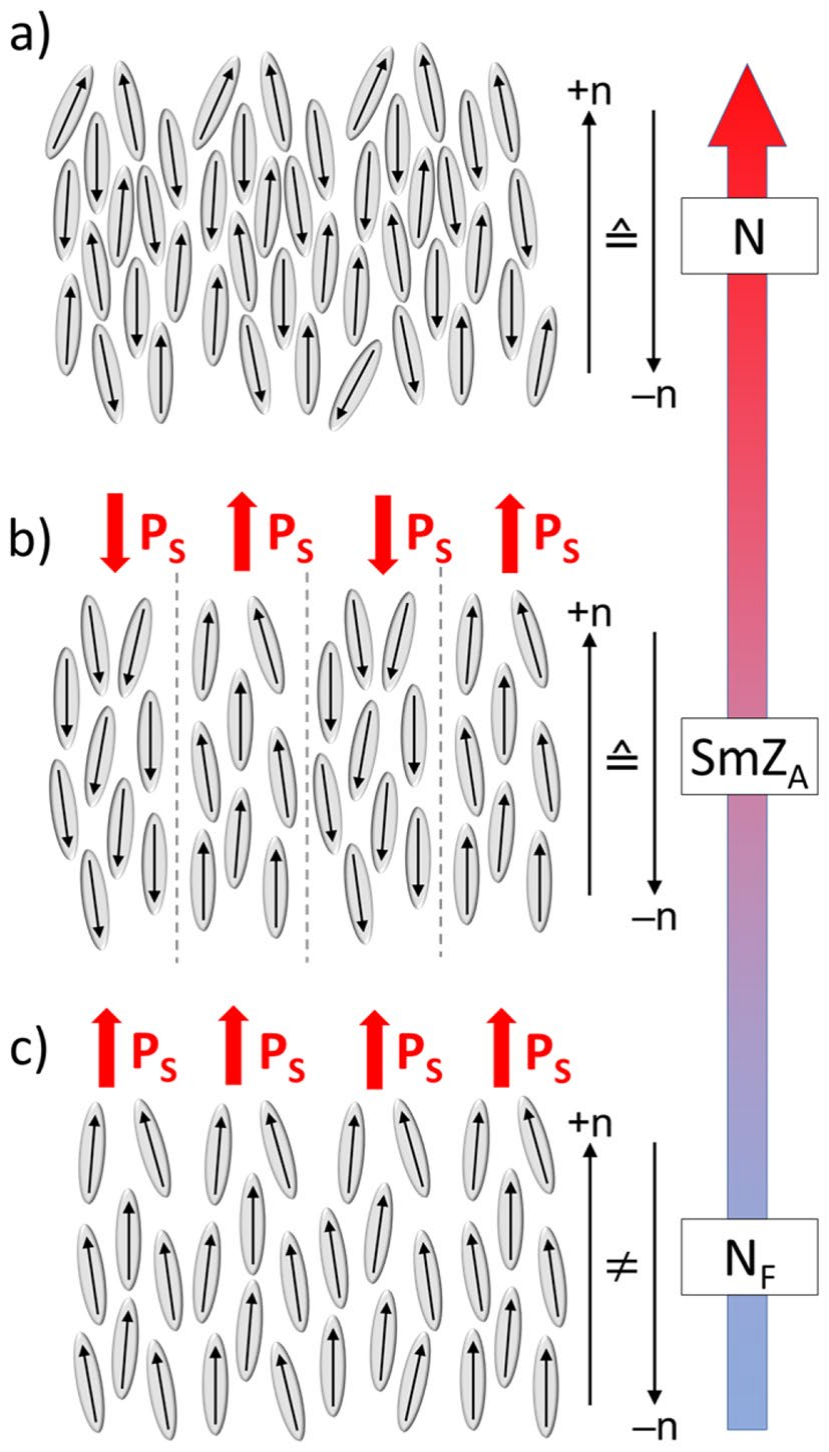
Schematic of the three liquid crystal phases—nematic N, smectic Z_A_ and ferroelectric nematic N_F_—found in AUUQU-2-N. The high-temperature nematic phase (**a**) is depicted with mesogens (grey) and their longitudinal dipole moments (indicated by arrows). Since on average half the dipoles point upwards and the other half downwards, the director **n** of the N phase is sign-invariant and the phase is paraelectric. (**b**) At lower temperature, the SmZ_A_ phase forms, with polar nematic layers with alternating directions of **P**_**s**_. Macroscopically, the sign invariance of **n** is preserved. (**c**) On further cooling, polar order develops and there is a transition to the ferroelectric nematic phase, the N_F_. This phase has broken sign invariance of **n** and a global spontaneous polarization **P**_**s**_ with a magnitude corresponding to near-perfect polar ordering of the molecular dipoles^7^.

This made it all the more surprising when in 2017, about 100 years after Born's publication, two highly polar mesogens, DIO^4^ and RM734^5–7^, were independently reported to form, in addition to an ordinary paraelectric nematic phase (N, see Fig. 1a), a second nematic, phase (N_F_, see Fig. 1c) that has spontaneous electric polarization, polar nematic order, and thus broken sign invariance of **n**. These discoveries have been followed by the publication of many examples of ferroelectric nematics^8–12^, including UUQU-4-N (compound 1 in reference^10^), which exhibits an N_F_ phase at room temperature. So far, most of these N_F_ mesogens are rod-shaped, non-centrosymmetric molecules with relatively extended (three- or four-ring) mesogenic cores, a single, relatively short, terminal (alkyl) chain, and highly polar substituents arranged to produce a large electric dipole moment (typically *µ* > 9 D) which coincides with the long molecular axis. The resulting spontaneous electric polarization in the N_F_ phase is typically of the order of several µC cm^–2^. It appears, however, that achieving polar ordering of the dipoles is a delicate balancing act, since even very small changes in the molecular structure can lead to the complete disappearance of the N_F_ phase^8^. The question of why certain mesogens form a polar N_F_ phase and other very similar mesogens do not thus remains a scientific challenge.

Further studies revealed that some of these N_F_ mesogens, in particular those of the DIO family, also form a third liquid crystal phase having antiferroelectric properties, observed between the N and N_F_ phases^13,14^. This phase has a 1D-modulated, antipolar structure of polar nematic layers in which the direction of local **P**_**s**_ alternates from layer to layer (see Fig. 1b). This antiferroelectric phase has low permittivity and its polarization reversal current exhibits a characteristic antiferroelectric double peak. Polarized light microscopy studies of planar glass cells reveal numerous optical effects and textural structures, including zig-zag defects, which show that this is a layered phase, and determine the layer normal and director orientation in the cells, confirming that in the SmZ_A_ phase, the polar nematic director is parallel to the layer planes. In the case of DIO, these observations are supported by non-resonant and resonant synchrotron-based X-ray diffraction studies, which revealed additional sharp Bragg-peaks that indicate the presence of well-defined density and polarization modulations having periodicities of respectively 9 and 18 nm^14^. The detection of Bragg peaks, together with the observation of characteristic zig-zag-wall textures, suggested the classification of this antiferroelectric phase as a new kind of smectic, the SmZ_A_, where “Z” indicates that the local director is parallel to the smectic layers (in contrast to the smectic A where the director is normal to the layers), and “A” indicates that the phase is antiferroelectric, with the polarization in adjacent layers alternating in direction. The periodic reversal of the polarization direction must be accompanied by a periodic density modulation, with the average electron density in the vicinity of the dashed lines in Fig. 1b, for example, being different from the electron density in the middle of the layers.

The splay instability of wedge-shaped mesogens leading to the 1D-periodic splay-nematic structure was proposed to explain the formation of quasi-periodic domains of opposite **P**_**s**_ observed in the N_F_ phase, where stripe-like domains with a modulation period of about ten microns were reported^15,16^. The exact nature of this proposed splay-nematic phase, and of its possible relationship to the SmZ_A_ with its much shorter 18 nm modulation period, is still unclear.

**Figure 2:**
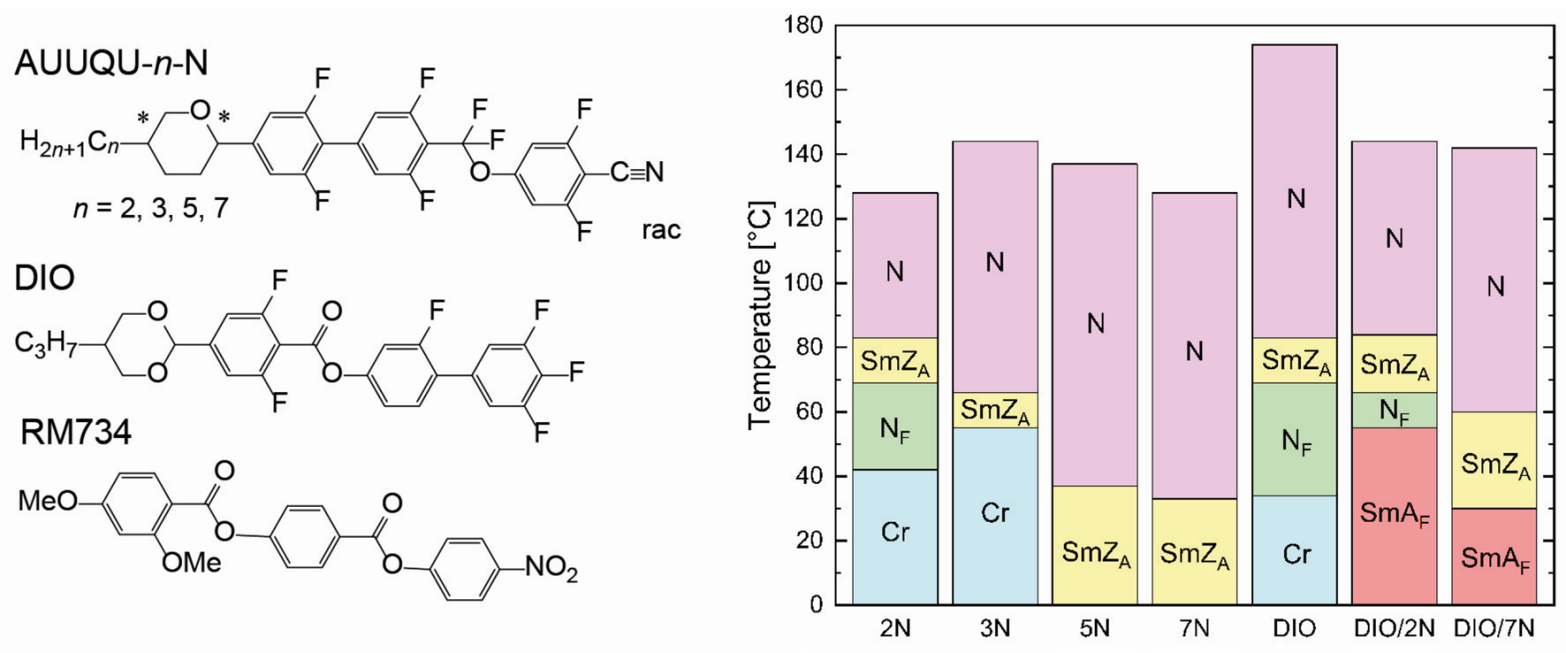
Structures of the AUUQU-*n*-N homologues and the archetypal ferroelectric materials DIO^4^ and RM734^5–7^, as well as phase sequences of the studied materials on cooling (denoted here as *n*N for brevity) compared to pure DIO and its mixtures with two of the homologues^17^. Longer alkyl chains stabilize the nematic phase (pink) and the antiferroelectric phase (yellow), while the ferroelectric nematic phase (green) is only observed for a chain length *n* = 2. For *n* > 3, crystallization (blue) is suppressed and the antiferroelectric phase freezes into a glassy state. The phase sequence of DIO is similar to that of AUUQU-2-N. The addition of DIO to AUUQU-2-N and AUUQU-7-N preserves their higher temperature phase sequences and leads, in addition, to the appearance of the ferroelectric smectic A phase (SmA_F_) at low temperatures.

**Figure 3:**
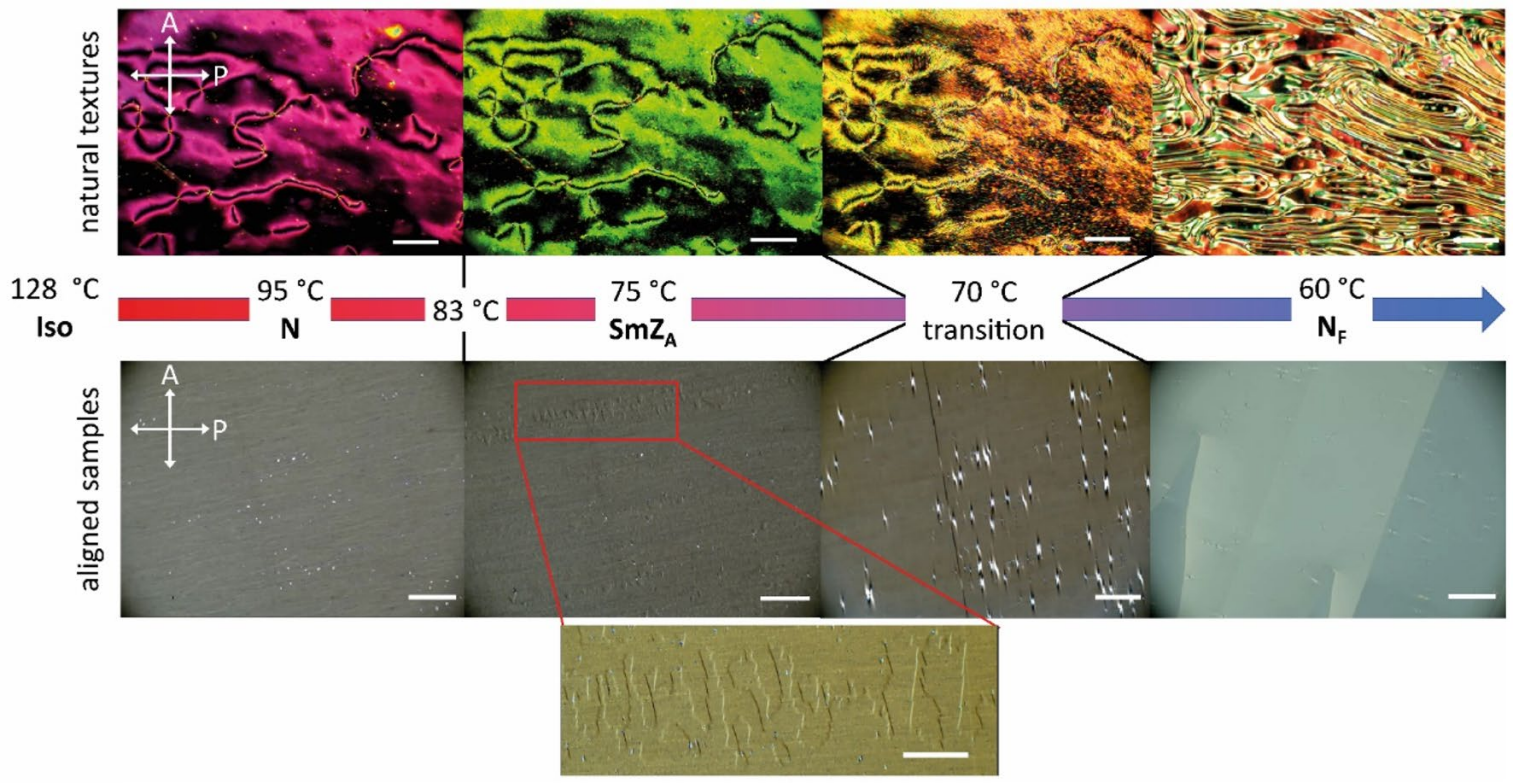
Optical textures of AUUQU-2-N on an untreated microscope slide with cover slip (top row) and in an LC cell coated with polyimide that is rubbed antiparallel on the two surfaces (bottom row). The nematic phase (from 127.6 to 83 °C) shows an ordinary schlieren texture with two- and four-armed defects. These defects stay in place and do not change shape on transitioning to the SmZ_A_ phase at 83 °C. The texture however becomes furry and the flickering associated with director fluctuations slows down. On further cooling to 70 °C, there is a transition to the N_F_ phase, with the defects disappearing and ferroelectric domain walls forming. In the rubbed cell, the nematic phase is well aligned. The alignment does not change on cooling into the SmZ_A_ phase but zig-zag defects (shown at higher magnification in the expanded view at bottom) characteristic of a chevron layer structure appear^20–22^. During the transition to the N_F_ phase, the zig-zag defects become brighter and are then replaced by mm-sized, polar monodomains with left- or right-handed twist^23^. (Expanded view scale bar: 100 µm; all other scale bars: 200 µm).

In this paper, we present a new series of ferro- and antiferroelectric nematic mesogens, the AUUQU-*n*-N homologs (Fig. 2). The molecular structure includes fluorinated building blocks, an oxane ring, and a terminal cyano group, all contributing to a large longitudinal molecular dipole moment of about 12.5 D. The mesogenic behavior of these compounds as pure materials and in mixtures with DIO is reported here. The shortest homolog, AUUQU-2-N, has three distinct liquid crystal phases, a common nematic N at high temperatures, a monotropic ferroelectric nematic N_F_ at low temperatures, and an antiferroelectric phase between the N and N_F_ phases. In the longer homologs, the N_F_ phase disappears in favor of a broader antiferroelectric phase which extends down to and below room temperature. Our previously published study^17^ has explored the thermotropic mesomorphism of 50:50 mixtures of DIO with AUUQU-2-N and with AUUQU-7-N. The AUUQU-2-N/DIO mixture exhibited a nematic (N)-smectic Z_A_ (SmZ_A_) – ferroelectric nematic (N_F_)-SmA_F_ phase sequence and AUUQU-7-N/DIO an N-SmZ_A_-SmA_F_ phase sequence, as indicated in Fig. 3.

Even though no sharp layer peaks have been observed to date in the x-ray diffraction patterns of the antiferroelectric phases of the pure AUUQU-*n*-N homologs, the textures seen in the microscope, including zig-zag-walls, and the positions of the antiferroelectric phases in the observed phase sequences, strongly suggest that these are further examples of the SmZ_A_ phase. We will discuss below the role of additional, short-range ordered structures including smectic C-type correlations (or "skewed cybotactic clusters") which we have observed in all three phases, N, SmZ_A_ and N_F_. Overall, this series of nematogens enriches the ferroelectric nematic realm, expanding the still rather limited pool of N_F_-materials and shedding new light on the understanding of antiferroelectric liquid crystal phases and the balance between polar and antipolar order.

## Results and discussion

### Studied materials

The chemical structures of the materials under investigation are depicted in Fig. 2 and compared to DIO and RM734. Mesogens of the so called AUUQU-*n*-N homologous series include fluorinated building blocks, an oxane ring and a terminal cyano group, all contributing to its strong molecular dipole moment. DFT-computations of AUUQU-2-N yield a dipole moment of around 12.5 D (supplementary Fig. S4), which is larger than DIO and RM734. In terms of chemical structure, the homologous series is more similar to DIO, due to the fluorination and the four-ringed structure. The oxane ring introduces chirality into the molecule but we used a racemic mixture in all experiments.

Several homologues of AUUQU-n-N were investigated but here, we focus on AUUQU-2-N as it has both the ferroelectric nematic N_F_ and the antiferroelectric SmZ_A_ phase. Comparative studies of the higher homologues are found in the Supporting Information.

### Phase sequences

The phase sequences of the homologous series, determined by a combination of differential scanning calorimetry (DSC, supplementary Fig. S2), POM, and SAXS, are shown in Fig. 2. The SmZ_A_ phase is observed in all of the homologues. AUUQU-2-N is the only material studied here that shows both the antiferroelectric SmZ_A_ and ferroelectric nematic N_F_ phases. It is thus the second example after DIO^4,13,18^ to exhibit a phase sequence of Iso-N-SmZ_A_-N_F_-Cr. If the phase sequences are compared, it can be seen that increasing the alkyl chain length stabilizes the nematic phase and destabilizes the N_F_. For a chain length of *n* $\ge$ 5, the SmZ_A_ phase is broad and stable down to room temperature, transitioning into a glassy state well below 0 °C.

The fact that only the *n* = 2 homologue exhibits the ferroelectric nematic phase can be understood in terms of the model of polar rods proposed by Madhusudana^19^: since in a polar nematic the mesogens are “locked” with their dipole moments in one direction, they need to have compatible surface charge-density waves. If the length of these waves changes, for example, as a result of changing the length of the molecule, this locking mechanism fails. The same holds true for DIO homologues, where a longer chain length suppresses the ferroelectric nematic phase and only the antiferroelectric phase remains^8^.

### Optical textures

Textures of AUUQU-2-N observed under a polarizing optical microscope (POM) are shown in Fig. 3. Both the "natural textures" on an untreated slide with cover glass and the planar-aligned textures in a rubbed liquid crystal cell are shown. Since the N_F_ and SmZ_A_ phases are of monotropic nature, the textures are viewed on cooling.

In an untreated cell (Fig. 3, top row), the paraelectric nematic phase obtained on cooling the LC from the isotropic exhibits a typical schlieren texture with half-integer and integer disclinations. On further cooling, a transition to the SmZ_A_ phase takes place which leaves the defects unchanged in appearance. The texture becomes fur-like and the optical flickering associated with Brownian fluctuations of the molecular orientation field more or less disappears, with only a very slow motion remaining. A few degrees lower, a transition to the N_F_ phase occurs, which this time does affect the defects, with the schlieren replaced by what resembles a fingerprint texture.

**Figure 4:**
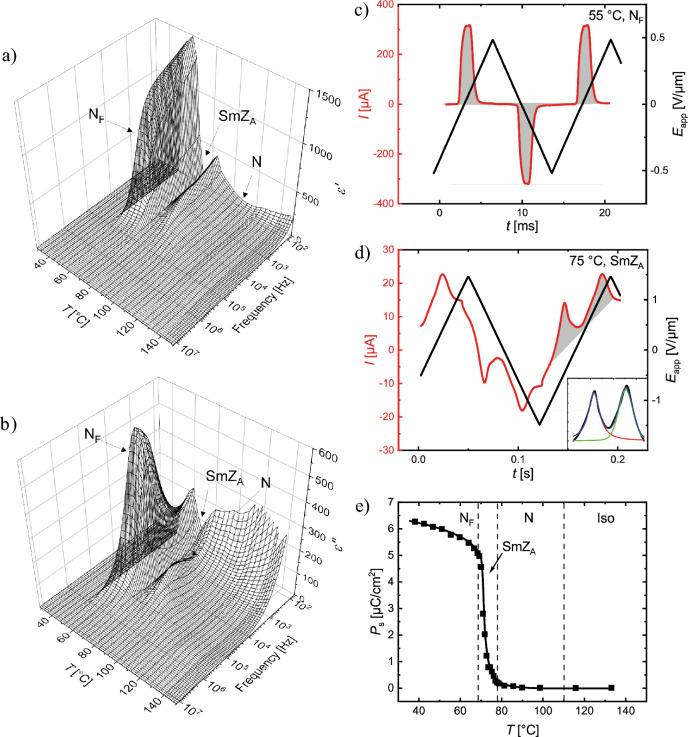
Dielectric and field-reversal measurements showing the dielectric properties, field response, and spontaneous polarization of AUUQU-2-N. Measurements of (**a**) the dielectric susceptibility $\varepsilon ^{\prime}$ and (**b**) the dielectric loss $\varepsilon ^{\prime}{^{\prime}}$ were carried out during cooling in 20 µm thick cells with polyimide and ITO coating. A relatively high $\varepsilon ^{\prime}$ can already be seen in the low-temperature nematic phase (**a**). Shortly before the transition to the SmZ_A_ phase, $\varepsilon ^{\prime}$ increases rapidly, which is likely due to the reduction of the splay elastic constant (an effect also observed in RM734^11,16,27^) and deviations from strict planar alignment. Following the transition to the SmZ_A_, $\varepsilon ^{\prime}$ falls rapidly, producing a deep notch between the N_F_ and N phases. In the N_F_ phase, $\varepsilon ^{\prime}$ increases again, into the thousands, which is typical of ferroelectric materials. Similar phenomena are seen in the measurements of $\varepsilon ^{\prime}{^{\prime}}$ (**b**). The relaxation frequency of the nematic phase starts in the kHz range and slows down on cooling. It approaches the MHz range as $\varepsilon ^{\prime}{^{\prime}}$ increases simultaneously. In the SmZ_A_ the frequency does not change significantly. Further cooling leads to a relaxation frequency in the low MHz range. The transition to lower frequencies is connected to a collective mode within the N_F_ phase, which must be slower than the nematic relaxation frequency. Polarization-reversal measurements in (**c**) the N_F_ and (**d**) the SmZ_A_ phases of AUUQU-2-N were carried out in 5-micron-thick planar-aligned cells with in-plane ITO electrodes. In the N_F_ phase, the current response (red) to a triangular applied field (black) shows a single peak (the integrated area is shown in grey). On transitioning into the SmZ_A_ phase, this current signal appears as two peaks with equal area, each corresponding to 3 µC/cm^2^ and adding up to the same total polarization value as in the N_F_ phase. The presence of two equal area peaks, shown in more detail in the inset of (**d**), confirms the antiferroelectric character of this phase. The temperature dependent polarization (**e**) shows zero polarization in the paraelectric nematic and increases slowly after transition into the SmZ_A_. This slow increase is due to the threshold voltage of the switching being temperature-dependent. In the N_F_ the polarization shows a saturation value of 6.25 µC/cm^2^. This polarization is near the computed theoretical maximum of 6.5 µC/cm^2^ if all the dipoles are perfectly aligned.

Entirely different textures are observed in a thin, planar-aligned LC cell (Fig. 3, bottom row). The ordinary nematic phase is aligned almost perfectly, with complete extinction between crossed polarizers achieved by rotating the sample. This alignment is maintained on cooling into the SmZ_A_ phase, with very little change in birefringence across the transition, as is the case in DIO and the AUUQU-2-N/DIO mixture^17^, but on cooling a few degrees, zig-zag defects^20,22^ appear, as shown in Fig. 4. These defects, which mediate changes in the folding direction of smectic layers in a chevron configuration^14,20,21^, were originally described by Clark et al. for the case of chiral smectic C phases^24^. The presence of the zig-zag defects is strong evidence for smectic ordering with the layers normal (or nearly normal) to the cell plates. The nematic director is oriented parallel to the cell plates, with the director parallel to the smectic layer planes, a key attribute of the SmZ_A_. On approaching the transition into the N_F_ phase, these zig-zag defects disappear and millimetre-sized domains of the N_F_ phase nucleate from bright point defects. Neighboring domains are observed to have opposite twist sense, which can be confirmed by uncrossing the polarizers (supplementary Fig. S5). The antiparallel rubbing direction of the cell surfaces forces the (polar) director **n** to perform a $\pi$-rotation from the top surface to the bottom^13,23,25^. Since right- and left-handed twist is energetically equivalent, both kinds of domains form, with roughly equal occurrence. As seen in Fig. 3, the overall phase sequences of pure DIO, 50%DIO/50%AUUQU-2-N, and pure AUUQU-2-N are very similar, with the exception of the appearance of the SmA_F_ phase in the mixture. This is likely a result of the strong structural similarity in molecular size and the magnitude and spatial distribution of the dipole moments of DIO and AUUQU-2-N, a resemblance which is apparently reduced in the longer AUUQU-*n*-N homologs. The similarity of the phase behavior in pure DIO, the DIO/AUUQU-2-N mixture, and pure AUUQU-2-N evident in Fig. 3, including a common SmZ_A_ range suggestive of ideal mixing behavior (~ 68 °C < *T* <  ~ 83 °C), is evidence that the SmZ_A_ is the same phase across the DIO/AUUQU-2-N composition range. Further evidence for this is the similarity of the SmZ_A_ layer spacings in pure DIO ( *d* = 8.8 nm) and in the 50%DIO/50%AUUQU-2-N mixture (*d* = 7.7 nm), in which comparison the SmZ_A_ phase of 50%DIO/50%AUUQU-7-N can be included ( *d* = 10.5 nm)^17^.

### Dielectric measurements

Dielectric measurements analyse the polarization response of materials to an external electric field. In the case of ferroelectric LC materials this response is unique, since the collective relaxation mode of the aligned dipoles gives rise to a huge imaginary dielectric permittivity $\varepsilon ^{\prime} {^{\prime}}$. It has recently been proposed by Clark et al.^26^ that, as a result, impedance measurements such as those carried out on ferroelectric nematic-filled cells do not describe the high capacitance of the ferroelectric material but rather its low resistance, the latter enabling the charging of the high-capacitance insulating layers (polyimide) at the interface of the LC and the electrodes. Nevertheless, dielectric measurements are relevant in proving the ferroelectric nature of polar nematic materials, where such an apparent large bulk material capacitance is due to the high polarization-mediated conductivity of the ferroelectric nematic phase.

The dielectric spectroscopy scans of AUUQU-2-N as a function of temperature are shown in Fig. 4a,b; measurements of the higher homologues are found in the SI (Fig. S3).

Starting at high temperatures in the nematic phase, a relatively high dielectric permittivity $\varepsilon ^{\prime}$ is observed. The permittivity increases continuously during cooling to values of up to 400 until just before the transition to the antiferroelectric phase. This increase has been proposed by Sebastián et al. to be caused by an increase in out-of-plane fluctuations of the director driven by a pre-transitional decrease in the splay elastic constant^11,15,16,27^. We suggest that this effect could be the result of a kind of Curie–Weiss pretransitional effect arising from the presence of the N_F_ phase at lower temperatures.

Immediately after transitioning into the SmZ_A_ phase, $\varepsilon ^{\prime}$ drops to around 100. This comparatively low permittivity confirms the antiferroelectric nature of this phase, where the global polarization is cancelled by the alternating orientation of local **P**_**s**_ directions. Finally, in the ferroelectric nematic phase, the apparent real permittivity component increases into the thousands, depending on cell thickness and alignment. This apparent giant dielectric response originates from the above mentioned dissipative collective reorientation of long-range correlated molecular dipoles in ferroelectric nematic materials. In addition, the increasing cooperativity of the dipoles—from the isotropic to the N_F_ phase—leads to a slowing down of the collective mode, which is best seen in the imaginary part of the permittivity $\varepsilon ^{\prime}{^{\prime}}$ (Fig. 4b). Here *R*, the low, polarization-mediated bulk resistance of the N_F_, which is in series with the large interfacial capacitance, *C*, gives a large time constant τ = *RC* that determines the angular frequency 1/τ of the relaxation. This is definitive evidence for fluid nematic ferroelectricity, complementing the observation of spontaneous electric polarization.

### Polarization reversal measurements

Ferroelectric materials exhibit a spontaneous electric polarization **P**_**s**_, whose direction can be reversed in an applied electric field. This reversal produces a distinct peak in the electric current, the measurement of which is used to determine the magnitude of **P**_**s**_. AUUQU-2-N samples were filled into LC cells with in-plane-switching (IPS) electrodes, a triangular voltage was applied, and the current response was measured. The current response is shown in Fig. 4c,d. A polarization of more than 6 µC/cm^2^ was measured in both the SmZ_A_ and N_F_ phases. This polarization is higher than in DIO and close to that of RM734^4,7,13,14^. The saturation value attained at low temperature is close to the theoretical maximum (6.5 µC/cm^2^) for AUUQU-2-N estimated using the density $\rho =1.4$ g/mol and the calculated dipole moment $\mu$ of the substance via ${P}_{S}=\left(\frac{\rho }{M}\right) {N}_{A} \mu$, with $M$ the molar mass and ${N}_{A}$ the Avogadro constant. This means that all dipole moments are essentially perfectly aligned in one direction along **n**.

The single current-reversal peak of the N_F_ phase (Fig. 4c) splits into two separate peaks in the antiferroelectric SmZ_A_ phase (Fig. 4d). These are characteristic of an antiferroelectric material, with each peak having the same area, reflecting two distinct switching processes: first from the ferroelectric state with **P**_**s**_ up to the antiferroelectric state and second from the antiferroelectric state to the ferroelectric state with **P**_**s**_ down. After baseline subtraction (see Fig. 4d, inset), the calculated area of each antiferroelectric current peak is seen to be half the area of the ferroelectric peak. However, the area as well as the shape of the two peaks of the SmZ_A_ phase are very frequency- and voltage-dependent. The apparent increase in magnitude of the **P**_**s**_ in the SmZ_A_ phase on cooling is an artefact of the temperature dependence of the degree of switching from the antiferroelectric to the ferroelectric state in an applied field of fixed amplitude. At higher temperatures, the switching is incomplete but as the sample approaches the N_F_ transition, the threshold voltage for switching from the antiferroelectric ground state into the induced ferroelectric state decreases, resulting in more complete switching and an apparent increase of **P**_**s**_.

### X-ray diffraction

**Figure 5:**
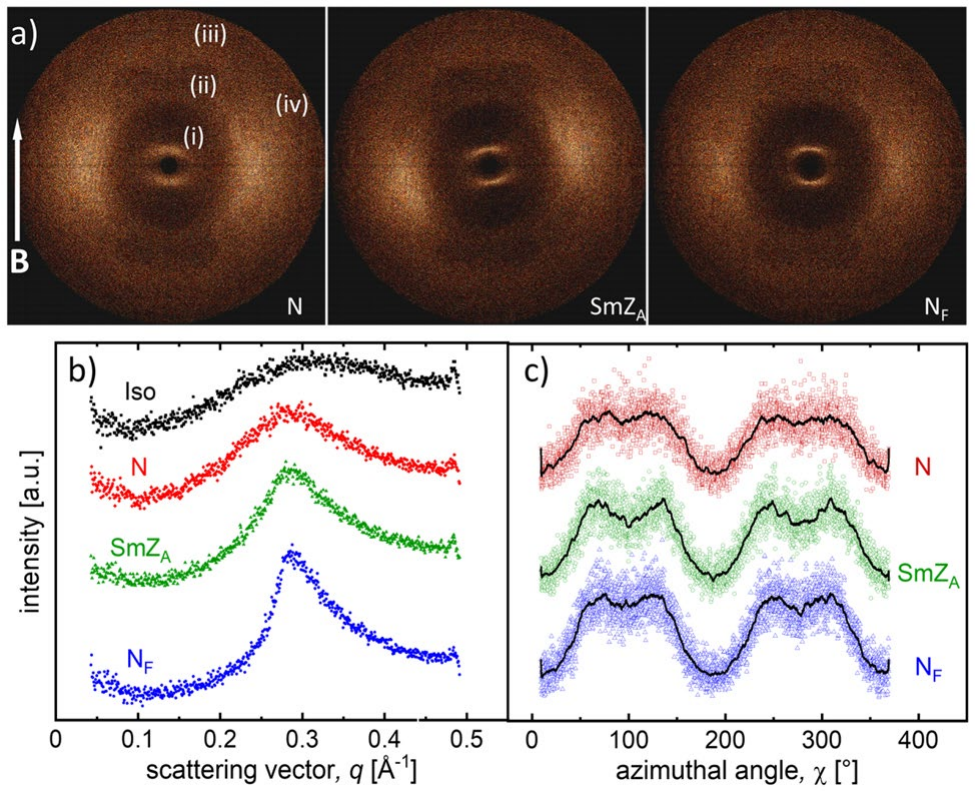
(**a**) The wide-angle X-ray scattering (2D WAXS) patterns of AUUQU-2-N aligned with a magnetic field **B** in different liquid crystalline phases (N: 101 °C, SmZ_A_: 74 °C, and N_F_: 64 °C) show only diffuse peaks. Along the meridian, three distinct signals are visible: (1) small-angle scattering (at q = 0.3 Å^−1^) associated with the end-to-end stacking of the mesogens, (2) (at q = 1.2 Å^−1^) and (3) (at q = 1.87 Å^−1^) which are both additional signals previously observed in all materials with the N_F_ or SmZ_A_ phase. Scattering is also observed along the equator (at q = 1.37 Å^−1^), corresponding to the side-to-side stacking of the molecules. (**b**) Radial scans of the small-angle scattering peak (i) integrated over the azimuthal angle χ. The peak is very diffuse in the nematic phase, due to the lack of a pronounced long-range positional order. As the sample is cooled to the SmZ_A_ and N_F_ phases, the scattering becomes slightly sharper and more intense but remains diffuse. (**c**) A complete azimuthal scan at *q* = 0.29 Å^−1^ integrated over a range of range $\delta$*q* =  ± 0.1 Å^−1^ indicates that the small-angle scattering peaks shown in (**b**) are not pure Lorentz curves in the azimuthal direction. Instead, they show a slight splitting, indicative of smectic C-type long-range correlations in all three phases, although the extent of this structure varies in the individual phases. The black line represents the average of the scattering. Similar splitting has been observed by Nishikawa et al. in mixtures of DIO^28^.

2D X-ray scattering studies were performed on magnetic-field aligned samples in all three liquid crystalline phases of AUUQU-2-N, with selected results shown in Fig. 5. Diffraction patterns of additional homologues are shown in Fig. S6 of the SI. The scattering patterns show several diffuse scattering arcs, three of them ((i), (ii) and (iii)) along the meridian and the fourth (iv) at the equator. The equatorial arc, with maximum at wavevector *q*_iv_ = 13.7 nm^–1^, originates from the typical wide-angle scattering from the side-by-side stacking of rod-shaped mesogens in a fluid phase.

The innermost diffuse arc (i), with maximum around *q*_i_ = 3.0 nm^–1^, comes from the end-to-end stacking correlations of the mesogens and becomes more intense and narrower with decreasing temperature in the SmZ_A_ and N_F_ phases, as seen in Fig. 5b. A closer look reveals that this diffuse signal is symmetrically split in azimuth $\upchi$ around the meridian by $\pm$ 30° (Fig. 5c), indicating the presence of short-range smectic C-fluctuations^28–33^ (also called "skewed cybotactic clusters"^30^). As seen in the azimuthal profiles in Fig. 5c, these fluctuations are present to varying extents in all three phases. In the case of the higher homolog AUUQU-5-N (Fig. S6), the splitting of the innermost maximum is even more pronounced.

In addition to the typical nematic scattering peaks (i) and (iv), two additional peaks ((ii) and (iii)) are observed along the meridian at q_iii_ = 0.12 Å^−1^ and q_iv_ = 0.187 Å^−1^, as shown in Fig. 5a. These peaks, which are characteristic of materials with N_F_ and/or SmZ_A_ phases^5,6,11,14,16,17,34^, are attributed to specific stacking modes along the long molecular axis^16,35,36^. It is interesting to note, however, that this scattering is temperature-independent and does not change sharpness or position during the phase transitions as seen in the Supporting Information (Fig. S8).

**Figure 6:**
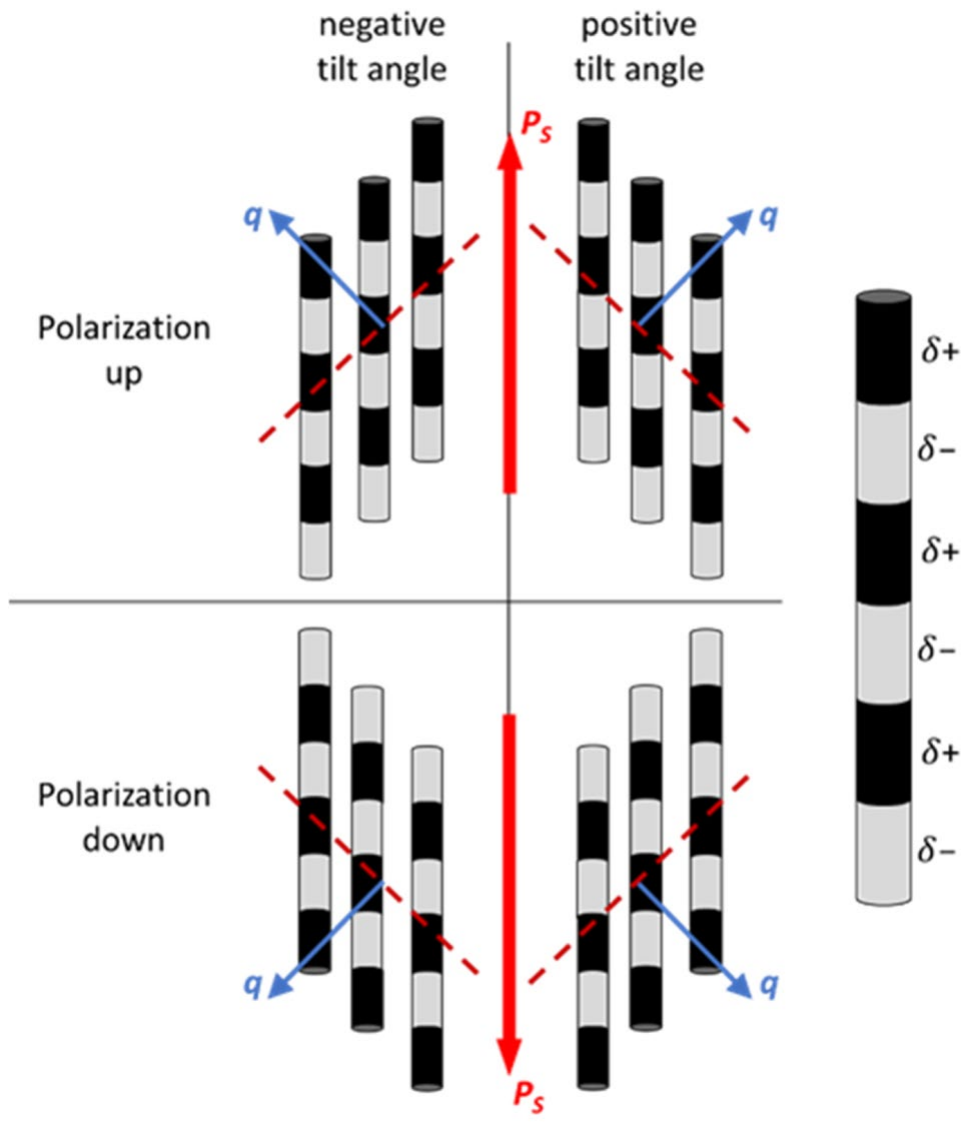
Schematic representation of a skewed cybotactic cluster in AUUQU-2-N. The mesogens are shown as rods with a surface charge-density wave, according to the proposed concept of Madhusudana^19^. To minimize the electrostatic interaction energy, the mesogens are slightly displaced with respect to each other. This displacement leads to four inclined scattering vectors **q** (blue) and azimuthal splitting of the X-ray pattern in the small-angle regime.

Molecular dynamics simulations by Mandle et al.^35,36^, as well as theoretical considerations by Madhusudana^19^, suggest that the side-by-side packing of two rod-shaped mesogens with their longitudinal dipoles parallel to each other requires a slight mutual displacement of the molecules in order to minimize their electrostatic interaction energy. This mutual displacement of nearest-neighbour molecules leads naturally to short-range SmC-like correlations (cf. Fig. 6), as observed in all three phases, N, SmZ_A_, and N_F_ in our X-ray experiments.

These findings support the underlying idea behind Madhusudana's model. Interestingly, the X-ray scattering shows that this essential packing motif is already present in the high-temperature paraelectric N phase. The presence of such small ferroelectric clusters explains the high dielectric susceptibility of the N phase shown in Fig. 4a.

**Figure 7:**
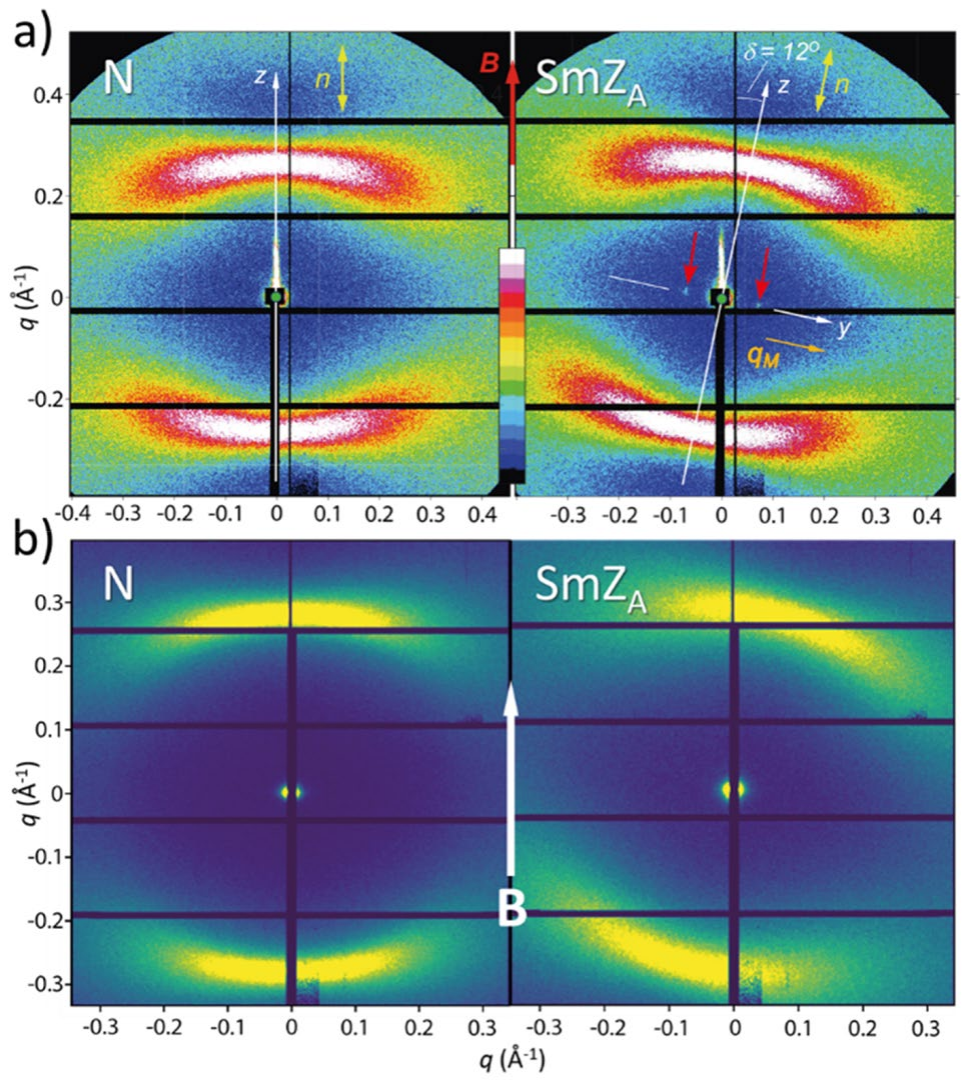
Small-angle x-ray scattering of (**a**) DIO^14^ and (**b**) AUUQU-2-N measured on the SMI microbeam line at NSLS II in the nematic and SmZ_A_ phases. The director alignment in the N phase is dictated by an external magnetic field B. However, in the SmZ_A_ a slight rotation is observed due to layer reorientations caused by a small reduction on layer thickness. The X-ray images all show characteristic scattering along the director, which is attributed to the end-to-end stacking of the molecules. The diffraction pattern of DIO in the SmZ_A_ phase shows faint Bragg peaks (red arrows) along the y-axis, corresponding to a *d*-spacing of 8.8 nm. These peaks imply a lamellar structure with the layer normal perpendicular to the director. No such peaks are detected in the SmZ_A_ phase of AUUQU-2-N.

While the optical experiments establish the general SmZ_A_ structure (layered with the director and polarization parallel to the layer planes) for the phase between the N and N_F_, they do not provide information on the smectic layer spacing. To address this issue, we performed non-resonant, synchrotron-based X-ray diffraction experiments, first on DIO^14^ and then on 50%AUUQU-2-N/50%DIO and 50%AUUQU-7-N/50%DIO mixtures^17^, as well as on the AUUQU-2-N single component as reported here. In these experiments, additional small but distinct Bragg scattering peaks could be observed at small *q* along the y-direction (the equatorial direction, normal to the director) at some temperatures and concentrations, as exemplified in Fig. 7a for the case of DIO. The intensity scans along *q*_y_ shown in Ref.^14^ are averages through a chosen range δ*q*_z_ of *q*_z_ that includes the entire peak height in that direction but no more, a choice that minimizes the background intensity and its photon counting noise relative to that of the peak. The Bragg peak positions then give the period of the electron density modulation of the smectic layering, enabling measurement of the SmZ_A_ layer spacing *d* in DIO (*d* = 8.8 nm)^14^, the AUUQU-2-N/DIO mixture (*d* = 7.7 nm), and the AUUQU-7-N/DIO mixture (*d* = 10.5 nm). The period of the polarization modulation is 2*d*, as confirmed by carbon-edge resonant X-ray scattering^14^.

In DIO, the SmZ_A_ layer reflections were observable in synchrotron-based SAXS experiments as peaks on background over the entire SmZ_A_ phase range, even appearing as weak, diffuse reflections in the neighbouring phases^14^. In the AUUQU-2-N/DIO and AUUQU-7-N/DIO mixtures, in contrast, peaks are observable over only a small part (3 to 5 °C) of the entire SmZ_A_ temperature range (Δ*T* ~ 15 °C in Fig. 3, as determined by the POM observations). At higher and lower temperatures, the peak is masked by the fluctuations in the background scattering. In the case of AUUQU-2-N and AUUQU-7-N, these SmZ_A_ peaks were not detected at any temperatures in the SmZ_A_ range, which again is determined by POM observations of structures such as zig-zag-wall textures clearly indicative of a smectic-like, layered structure.

Another distinctive feature of the SAXS images of pure DIO, of the AUUQU-2-N/DIO and AUUQU-7-N/DIO mixtures, and of pure AUUQU-2-N, is an obvious rotation in the SmZ_A_ phase of the entire 2D SAXS pattern, with examples from pure DIO and of pure AUUQU-2-N shown in Fig. 7a,b, respectively. Such reorientation is evidence for the tilting of systems of fluid smectic layers in chevron-type defects^14^, providing further confirmation that the intermediate phase in pure AUUQU-2-N has a smectic-like structure.

**Figure 8:**
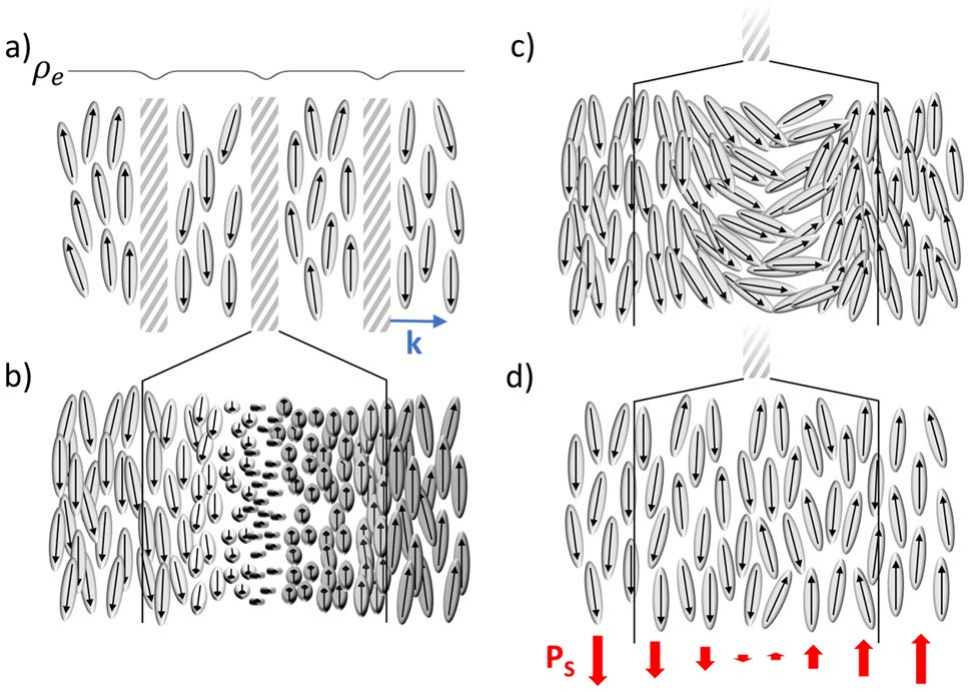
(**a**) In a schematic representation of the SmZ_A_ phase, a lamellar structure with polar molecules oriented parallel to the layers is shown. Each layer is polar but the polarization is cancelled globally by the opposing signs of polarization in adjacent layers. The variation of the electron density along the layer normal **k**, depicted above the layer structure, remains essentially constant except at the transition from one layer to the next where there is a small change. If the contrast of the electron density at the layer boundaries is high enough, this modulation results in an equatorial X-ray signal at small *q*. The degree of contrast in the electron density depends on how the spontaneous electric polarization (**P**_**s**_) changes from layer to layer. If the polarization reorients via twist, forming a Bloch wall (**b**), or bend, forming a Néel wall (**c**), the deformation of the director field results in less efficient space-filling and a lower electron density at the layer boundaries. These structures appear to be ruled out by the optical observations. However, the transition from layer to layer can also be achieved without distorting the director field (**d**), by gradually increasing the fraction of molecular dipoles pointing in the direction opposite to the polarization in the starting layer, until finally a complete reversal of the preferred orientation occurs in the next layer. To a first approximation, this particular form of polarization reversal, called pure polarization reversal (PPR)^7^, effectively implies the presence of a non-polar nematic slab sandwiched between fluid, ferroelectric nematic-like layers of opposite polarization.

Since layers with opposite directions of **P**_**s**_ contribute the same electron density ${\uprho }_{e}\left(k\right)$ along the stacking direction **k** (see Fig. 8a), this periodic structure is detected in an X-ray experiment only if there is some variation in electron density at the interface between two adjacent layers. These interlayer boundaries with a reduced ${\uprho }_{e}\left(k\right)$ are indicated in Fig. 8a as gray shaded areas. Three idealized scenarios for the reversal of **P**_**s**_ and the subsequent variation in ${\uprho }_{e}\left(k\right)$ at the inter-layer boundaries are depicted in Fig. 8b–d. In the first two cases, the reversal of **P**_**s**_ is connected to a twist- or bend-deformation of the local director (Fig. 8b,c). These two scenarios are analogous, respectively, to the Bloch and Néel walls found in ferromagnets. It seems reasonable to assume that in these two cases the electron density is slightly lower because the packing density of the molecules (and thus the electron density) is somewhat reduced as a consequence of the elastic deformation. However, for a typical SmZ_A_ layer spacing, say *d* ~ 10 nm as found in DIO and the AUUQU-2-N/DIO and AUUQU-7-N/DIO mixtures, structures (b) and (c) are ruled out by comparing the birefringence of the SmZ_A_ to that of fully polar N_F_ or SmA_F_ phases^17^. The transition of homogeneous monodomains of the antiferroelectric AUUQU-7-N/DIO SmZ_A_, each of which is filled with a linear array of polarization reversal defects spaced by 10 nm^14^, to the fully polar ferroelectric SmA_F_, which has only a few such defects $(d \to \infty )$ is shown in Figs. 3C1 and 3C2 of Ref.^17^. Remarkably, these POM images show directly that this transition occurs with essentially *no visible change in birefringence*. However, taking the model in (c), for example, and dividing a *d* = 10 nm SmZ_A_ layer into four 2.5 nm-wide segments, with one segment being the splay-bend wall and three others having **P**_**s**_ vertical, we see that that the splay-bend segment effectively cancels the birefringence of one of the other segments, leaving the net birefringence contribution from only two of the four segments. That is, the SmZ_A_ would start out with only half the birefringence of a fully aligned SmA_F_ or N_F_, a huge difference, not observed in DIO or DIO/AUUQU-7-N mixtures, where the experimentally observed difference in birefringence value is tiny, around 0.001. By the same argument the structure in (b) would have one segment with low birefringence, giving ~ 0.75 of the birefringence of a fully aligned SmA_F_ or N_F_, also implying a huge difference that is again much larger than observed in DIO or DIO/AUUQU-7-N mixtures. These two cases show that, starting from the orientation within the domains that provides maximum birefringent phase shift for a vertical optic axis, reorientation of any sort through the domain wall will reduce this phase shift within the domain wall, and therefore reduce the average birefringence. As noted above, the observed birefringence is little changed from its uniform N_F_ value. A reasonable inference from this observation is that polarization reversal is achieved without reorientation through the domain walls, which is the case in Fig. 8d, showing "pure polarization reversal" (PPR) walls^7^, in which the nematic director field is nearly uniform, not requiring any elastic deformation. Instead, when passing from a SmZ_A_ layer with given polarization direction to the next, the fraction of molecules with oppositely directed longitudinal dipole moment increases continuously along **k** until the polarization direction is completely reversed. The structure at the wall midplane is that of a non-polar nematic, which experiment^37^ and simulation^11^ show to have a lower mass and therefore lower electron density than the polar N_F_-like parts of the structure (the interiors of the layers), such that an array of PPR walls, as in a SmZ_A_ phase, can be expected to produce non-resonant Bragg SAXS scattering. This and the uniformity of the director field, which gives the SmZ_A_ and uniformly polar phases nearly the same birefringence, makes the PRR wall array the likely scenario for the local SmZ_A_ structure^14^.

The correlation between Bragg peak SAXS intensity and the concentration of DIO in the 50:50 mixtures with AUUQU-2-N and AUUQU-7-N implies that having less DIO and more AUUQU-2-N or AUUQU-7-N in the sample leads to weaker SAXS Bragg scattering (lower peak intensity) from the SmZ_A_ periodicity. Given the already substantial difference in scattering intensity of the 100% DIO and 50% DIO samples, the absence of SAXS peaks in pure AUUQU-2-N and AUUQU-7-N is not too surprising, and points to smaller electron-density contrast in the domain walls in samples with less DIO.

Finally, we address the nature of the translational order in the SmZ_A_ phase. In ordinary smectics, where the layers are defined by a continuous, periodic variation in mass density, there is quasi-long-range translational order of the molecular centers in one dimension. In the SmZ_A_ phase, however, the layers appear to be essentially homogeneous, with little variation of the mass density within each layer. The variation of electron density that leads to Bragg scattering occurs principally across the paraelectric nematic slabs forming the interfaces between the polar layers. There are other examples in the LC literature where the periodicity of the phase is determined by a lower-symmetry interface between ordered states, such as the 3D defect network of the blue phase^38^ or the solvent layers in swollen smectics^39,40^.

## Conclusions

In this work, we introduced a new homologous series of nematogens showing polar and antipolar phases. These materials represent an important addition to the rather limited pool of such compounds known at this time and the studies of their phases, properties and structures provided some remarkable findings:

- In addition, to the common paraelectric nematic phase, a ferroelectric nematic phase and antiferroelectric phases are found. Interestingly, the antiferroelectric phase is the dominating phase in this series, while the ferroelectric nematic phase is only found in the shortest homologue *n* = 2.
- The ferroelectric nematic phase of AUUQU-2-N is rather similar to the archetypical cases in DIO and RM734. The material exhibits nearly the same phases and transition temperatures as DIO, as well as nearly perfect order of the longitudinal dipole moments leading to a saturation **P**_**S**_ at low temperature of 6.25 µC cm^–2^.
- In higher homologues, crystallization is prevented and the monotropic antiferroelectric phase is stable for hours, even at room temperature.
- The observed antiferroelectric phases are new examples of the recently reported SmZ_A_ phase, as they all show characteristic zig-zag wall textures.
- However, in contrast to the SmZ_A_ phase in pure DIO and a 50:50 mixture of DIO and AUUQU-2-N, we do not observe a sharp X-ray peak associated with the period of antipolar order in the antiferroelectric phase of AUUQU-2-N. We conclude that while this peak must necessarily appear in all SmZ_A_ phases, it is weaker in AUUQU-2-N. We suggest that the domain walls between the polar smectic layers provide less x-ray contrast in the absence of DIO.
- The similarities evident in Fig. 3 in the phase behavior and transition temperatures of DIO and AUUQU-2-N, the AUUQU-*n*-N homolog closest in structure to DIO, strongly suggest that the pure materials share the same phases.
- In the X-ray experiments, we observe short-range SmC-type correlations in all three phases—N, SmZ_A_ and N_F_—of the order of the molecular length. These correlations are consistent with the Madhusudana model of polar ordering in nematics. Interestingly, this essential packing motif is already established in the high-temperature N phase and explains the high dielectric susceptibility of the paraelectric N phase.

We are confident that these findings will shed new light on the understanding of polar and antipolar order in the ferroelectric nematic realm.

## Materials and methods

### General

The liquid-crystal materials that were studied here (AUUQU-n-N) were synthesized by Merck Electronics KGaA after the patent DE 103 53 658 A1 2004 06 09.

Computations of the molecular dipole moments were performed using the Gaussian 16 programs^41^ with the 6-31G(d) basis set and the B3LYP hybrid DFT-functional^42,43^.

### Microscopic studies

Phase transition temperatures, textures and alignment were investigated with a Leica DM 2700 P polarized light microscope, equipped with an Instec heating stage HCS302 controlled by an mk1000 temperature unit. The pictures were taken using a pixeLink video camera.

Natural textures were observed on an untreated microscope slide with a cover slip on top. Aligned samples were prepared in 1.6 µm thick LC cells (from the Military University of Technology in Poland) coated with polyimide, where the director **n** was aligned along the rubbing direction with no observable pre-tilt.

### X-ray diffraction

The samples were filled into a 0.7 mm diameter Mark capillary with an outer diameter of 0.01 mm (Hilgenberg glass No. 14). The director **n** was aligned with an external magnetic field (0.7 T) oriented perpendicular to the beam.

2D X-ray measurements were performed with a Bruker AXS NanoSTAR system (CuK_α_ radiation wavelength = 1.5418 Å, Goebel mirror monochromator, 0.1 mm point beam collimator, VÅNTEC-500 detector), equipped with a temperature-controlled sample holder cooled by a Unichiller from Huber. A sample detector distance of about 10 cm was used with an average recording time of one hour.

The synchrotron-based X-ray measurements were performed on the SMI beamline (12-ID) at NSLS II with a photon energy of 16.1 keV (wavelength = 0.7009 Å) and a beam size of 2 µm × 25 µm. Scattered intensity was recorded with a Pilatus3 1M detector positioned 2 meters from the sample.

### Spontaneous polarization reversal measurements

To analyze the spontaneous polarization the materials were filled in 5 µm thick, planar aligning LC cells from the Military University of Technology, Warsaw, coated with polyimide and rubbed perpendicular to the interdigitating in-plane ITO electrodes. The active electrode area was 5 cm by 5 cm with 10 µm thick electrodes and a gap of 20 µm. The samples were filled in the isotropic state by capillary action.

The spontaneous electric polarization was obtained from recording the current response to a triangular AC voltage with a frequency of 7–100 Hz. The voltage was generated with a 33500B waveform generator from KEYSIGHT and amplified with a 7500 amplifier from Krohn-Hite. The current response measured via a 0.5 k$\Omega$ resistor was amplified, noise reduced with a SR560 Low-Noise preamplifier from STANFORD RESEARCH SYSTEMS and afterwards recorded with a DSO-X 2004A Oscilloscope from KEYSIGHT.

### Dielectric measurements

The samples were filled in 20 µm thick sandwich cells from the Military University of Technology, Warsaw coated with polyimide and rubbed on both sides parallel. The cells have an ITO coating with an active area of 5 cm by 5 cm which was then connected to the analyzer. The dielectric response was measured with a 4192A LF Impedance Analyzer from Hewlett-Packard, with an oscillator amplitude of voltage of 0.1 Vrms. The temperature was controlled by an NOVOTHERM from Novocontrol.

### Dynamic scanning calorimetry

The samples of 3–5 mg were filled into PerkinElmer aluminum pans (part no. B016-9321). The measurement was done with a DSC8000 instrument from PerkinElmer. Heating/cooling rates of 1, 5, 10 and 50 K min^−1^ were used with three cycles each. For analysis, the first cycle was discarded and the software Pyris was used.

### Supplementary Information

Supplementary Figures.

## Data Availability

The datasets generated and/or analyzed during the current study are available from the corresponding author on request.

## References

[1] Born, M. Ueber anisotrope Flüssigkeiten: Versuch einer Theorie der flüssigen Kristalle und des elektrischen Kerr-Effekts in Flüssigkeiten. *Sitzungsber. Preuss. Akad Wiss.* **30**, 614–650 (1916).

[2] Kléman, M. Points, lines and walls. In* Liquid Crystals, Magnetic Systems and Various Ordered Media* (Wiley, 1983).

[3] Maier, W. & Saupe, A. Eine einfache molekular-statistische theorie der nematischen kristallinflüssigen phase. *Teil l. Z. Naturforsch. A* **14**, 882–889 (1959). 10.1515/zna-1959-1005

[4] Nishikawa, H. *et al.* A fluid liquid-crystal material with highly polar order. *Adv. Mater.* **29**, 1702354 (2017). 10.1002/adma.201702354 29023971

[5] Mandle, R. J., Cowling, S. J. & Goodby, J. W. A nematic to nematic transformation exhibited by a rod-like liquid crystal. *Phys. Chem. Chem. Phys.* **19**, 11429–11435 (2017). 28422219 10.1039/C7CP00456G

[6] Mandle, R. J., Cowling, S. J. & Goodby, J. W. Rational design of rod-like liquid crystals exhibiting two nematic phases. *Chem. Eur.* **23**, 14554–14562 (2017). 10.1002/chem.201702742 PMC5656819 28850751

[7] Chen, X. *et al.* First-principles experimental demonstration of ferroelectricity in a thermotropic nematic liquid crystal: Polar domains and striking electro-optics. *PNAS* **117**, 14021–14031 (2020). 32522878 10.1073/pnas.2002290117 PMC7322023

[8] Mandle, R. J. A new order of liquids: Polar order in nematic liquid crystals. *Soft Matter* **18**, 5014–5020 (2022). 35776092 10.1039/D2SM00543C

[9] Song, Y. *et al.* Development of emergent ferroelectric nematic liquid crystals with highly fluorinated and rigid mesogens. *Phys. Chem. Chem. Phys.* **24**, 11536–11543 (2022). 35506891 10.1039/D2CP01110G

[10] Manabe, A., Bremer, M. & Kraska, M. Ferroelectric nematic phase at and below room temperature. *Liq. Cryst.* **48**, 1079–1086 (2021). 10.1080/02678292.2021.1921867

[11] Mandle, R. J., Sebastián, N., Martinez-Perdiguero, J. & Mertelj, A. On the molecular origins of the ferroelectric splay nematic phase. *Nat. Commun.* **12**, 4962 (2021). 34400645 10.1038/s41467-021-25231-0 PMC8367997

[12] Li, J. *et al.* Development of ferroelectric nematic fluids with giant-ε dielectricity and nonlinear optical properties. *Sci. Adv.* **7**, eab5047 (2021). 10.1126/sciadv.abf5047 PMC8059932 33883139

[13] Brown, S. *et al.* Multiple polar and non-polar nematic phases. *Chemphyschem* **22**, 2506–2510 (2021). 34623724 10.1002/cphc.202100644

[14] Chen, X. *et al.* The smectic ZA phase: Antiferroelectric smectic order as a prelude to the ferroelectric nematic. *PNAS* **120**, e2217150120 (2023). 36791101 10.1073/pnas.2217150120 PMC9974471

[15] Sebastián, N. *et al.* Ferroelectric-ferroelastic phase transition in a nematic liquid crystal. *Phys. Rev. Lett.* **124**, 37801 (2020). 10.1103/PhysRevLett.124.037801 32031856

[16] Mertelj, A. *et al.* Splay nematic phase. *Phys. Rev. X* **8**, 41025 (2018).

[17] Chen, X. *et al.* Observation of a uniaxial ferroelectric smectic A phase. *PNAS* **119**, e2210062119 (2022). 36375062 10.1073/pnas.2210062119 PMC9704750

[18] Chen, X. *et al.* Ideal mixing of paraelectric and ferroelectric nematic phases in liquid crystals of distinct molecular species. *Liq. Cryst.* **49**, 1531–1544 (2022). 10.1080/02678292.2022.2058101

[19] Madhusudana, N. V. Simple molecular model for ferroelectric nematic liquid crystals exhibited by small rodlike mesogens. *Phys. Rev. E* **104**, 14704 (2021). 10.1103/PhysRevE.104.014704 34412337

[20] Rieker, *et al.* “Chevron” local layer structure in surface-stabilized ferroelectric smectic-C cells. *Phys. Rev. Lett.* **59**, 2658–2661 (1987). 10035615 10.1103/PhysRevLett.59.2658

[21] Ouchi, Y. *et al.* Smectic C* chevron layer structure studied by x-ray diffraction. *JJAP* **27**(5A), L725 (1988). 10.1143/JJAP.27.L725

[22] Handschy, M. A. & Clark, N. A. Structures and responses of ferroelectric liquid crystals in the surface-stabilized geometry. *Ferroelectrics* **59**, 69–116 (1984). 10.1080/00150198408240738

[23] Rudquist, P. Revealing the polar nature of a ferroelectric nematic by means of circular alignment. *Sci. Rep.* **11**, 24411 (2021). 34949781 10.1038/s41598-021-04028-7 PMC8702550

[24] Shao, R. F., Willis, P. C. & Clark, N. A. The field induced stripe texture in surface stabilized ferroelectric liquid crystal cells. *Ferroelectrics* **121**, 127–136 (1991). 10.1080/00150199108217616

[25] Chen, X. *et al.* Polar in-plane surface orientation of a ferroelectric nematic liquid crystal: Polar monodomains and twisted state electro-optics. *PNAS* **118**, e2104092118 (2021). 34050028 10.1073/pnas.2104092118 PMC8179187

[26] Clark, N. A., Chen, X., Maclennan, J. E. & Glaser, M. A. Dielectric spectroscopy of ferroelectric nematic liquid crystals: Measuring the capacitance of insulating interfacial layers.

[27] Sebastián, N., Mandle, R. J., Petelin, A., Eremin, A. & Mertelj, A. Electrooptics of mm-scale polar domains in the ferroelectric nematic phase. *Liq. Cryst.* **48**, 2055–2071 (2021). 10.1080/02678292.2021.1955417

[28] Nishikawa, H. *et al.* Nano-clustering mediates phase transitions in a diastereomerically-stabilized ferroelectric nematic system. *Commun. Mater.* **3**, 89 (2022). 10.1038/s43246-022-00312-9

[29] Saha, R. *et al.* Multiple ferroelectric nematic phases of a highly polar liquid crystal compound. *Liq. Cryst.* **49**, 1784–1796 (2022). 10.1080/02678292.2022.2069297

[30] Ghilardi, M., Adamo, F. C., Vita, F., Francescangeli, O. & Domenici, V. Comparative 2H NMR and X-ray diffraction investigation of a bent-core liquid crystal showing a nematic phase. *Crystals* **10**, 284 (2020). 10.3390/cryst10040284

[31] Nishiya, W., Takanishi, Y., Yamamoto, J. & Yoshizawa, A. Molecular design for a cybotactic nematic phase. *J. Mater. Chem. C* **2**, 3677–3685 (2014). 10.1039/C4TC00001C

[32] Hong, S. H. *et al.* Short-range smectic order in bent-core nematic liquid crystals. *Soft Matter* **6**, 4819 (2010). 10.1039/c000362j

[33] Francescangeli, O. *et al.* Ferroelectric response and induced biaxiality in the nematic phase of bent-core mesogens. *Adv. Funct. Mater.* **19**, 2592–2600 (2009). 10.1002/adfm.200801865

[34] Mandle, R. J., Cowling, S. J. & Goodby, J. W. Structural variants of RM734 in the design of splay nematic materials. *Liq. Cryst.* **48**, 1780–1790 (2021). 10.1080/02678292.2021.1934740

[35] Mandle, R. J. Implementation of a cylindrical distribution function for the analysis of anisotropic molecular dynamics simulations. *PloS One* **17**, e0279679 (2022). 36584026 10.1371/journal.pone.0279679 PMC9803122

[36] Mandle, R. J. In silico interactome of a room-temperature ferroelectric nematic material. *Crystals* **13**, 857 (2023). 10.3390/cryst13060857

[37] Parton-Barr, C., Gleeson, H. & Mandle, R. Room-temperature ferroelectric nematic liquid crystal showing a large and divergent density. *Soft Matter* **20**, 672–680 (2023). 10.1039/D3SM01282D 38164818

[38] Collings, P. J. *Liquid Crystals. Nature’s Delicate Phase of Matter* 2nd edn. (Princeton University Press, 2002).

[39] Murase, M., Takanishi, Y., Nishiyama, I., Yoshizawa, A. & Yamamoto, J. Hyper swollen perfluorinated smectic liquid crystal by perfluorinated oils. *RSC Adv.* **5**, 215–220 (2015). 10.1039/C4RA12938E

[40] Bruckner, J. R., Porada, J. H., Dietrich, C. F., Dierking, I. & Giesselmann, F. A lyotropic chiral smectic C liquid crystal with polar electrooptic switching. *Angew. Chem. Int. Ed.* **52**, 8934–8937 (2013). 10.1002/anie.201303344 23922272

[41] Frisch, M. J. *et al.**Gaussian 16* (Gaussian Inc., 2016).

[42] Becke, A. D. Density-functional thermochemistry. III. The role of exact exchange. *J. Chem. Phys.* **98**, 5648–5652 (1993). 10.1063/1.464913

[43] Chai, J.-D. & Head-Gordon, M. Long-range corrected hybrid density functionals with damped atom-atom dispersion corrections. *Phys. Chem. Chem. Phys.* **10**, 6615–6620 (2008). 18989472 10.1039/b810189b

