# Supplementary material for: New examples of ferroelectric nematic materials showing evidence for the antiferroelectric smectic-Z phase
Source: Sci Rep. 2024 Feb 23;14:4473. doi: 10.1038/s41598-024-54832-0 (PMC11319781; doi:10.1038/s41598-024-54832-0)
Supplement: Supplementary file 1 — Supplementary Figures. [file 41598_2024_54832_MOESM1_ESM.pdf]

New examples of ferroelectric nematic materials showing evidence for the antiferroelectric smectic-Z phase

Pierre Nacke[1], Atsutaka Manabe[2,3], Melanie Klasen-Memmer[2], Xi Chen[4], Vikina Martinez[4], Guillaume Freychet[5], Mikhail Zhernenkov[5], Joseph E. Maclennan[4], Noel A. Clark[4], Matthias Bremer[2], Frank Giesselmann[1*]

[1]Institute of Physical Chemistry, University of Stuttgart, 70569 Stuttgart, Germany; [2]Merck Electronics KGaA, Display Solutions, 64293 Darmstadt, Germany; [3]Individual researcher (since 01.01.22), 64625 Bensheim, Germany; [4]Dept. of Physics and Soft Materials Research Center, University of Colorado, Boulder, CO 80309, USA;[5]Brookhaven National Laboratory, National Synchrotron Light Source-II, Upton, NY 11973, USA

Corresponding author:
Prof. Dr. Frank Giesselmann, Institute of Physical Chemistry, University of Stuttgart, Pfaffenwaldring 55, 70569 Stuttgart, Germany, email: f.giesselmann@ipc.uni-stuttgart.de

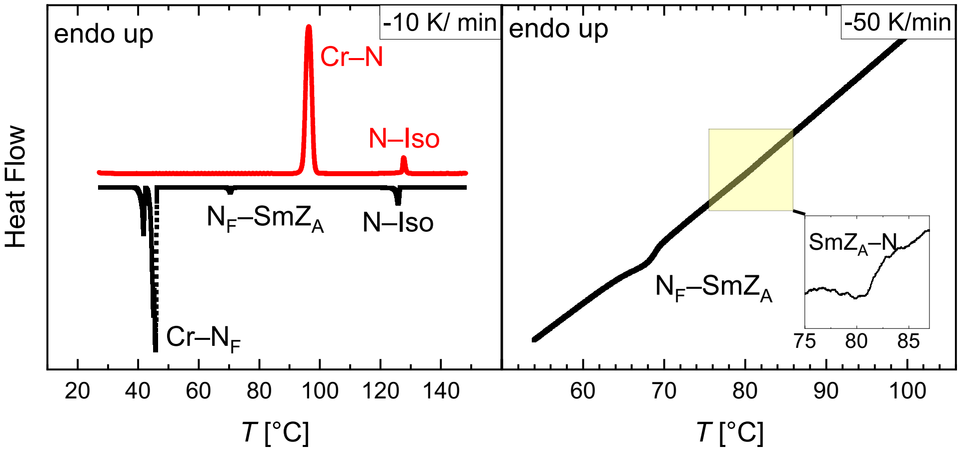

Figure S1: Differential scanning calorimetry of AUUQU-2-N measured with cooling rates of (a) -10 K min^{-1} and (b) -50 K min^{-1}. The signal corresponding to the transition from N to SmZ$_A$ only becomes observable after scanning with a high cooling rate. As a result of the monotropic nature of both the N$_F$ and SmZ$_A$ phase, these phases only appear in the cooling process.

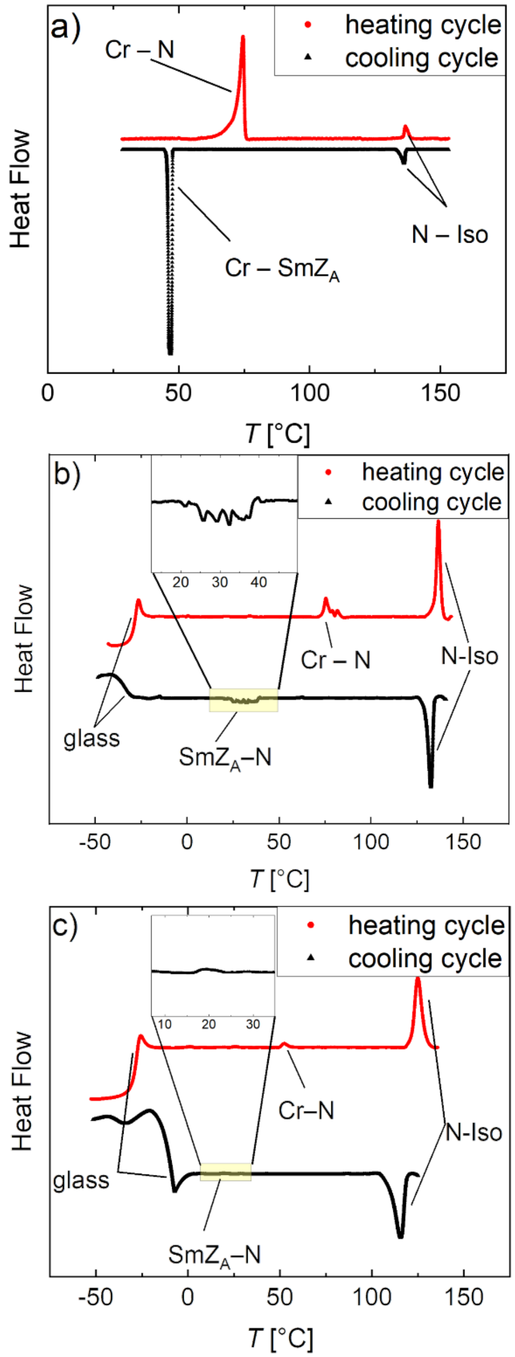

Figure S2: Differential scanning calorimetry of AUUQU-*n*-N (*n* = 3, 5, 7; (a) –(c)) measured with a cooling rate of -10 K min^{-1}. In (a) AUUQU-3-N the transition into the antiferroelectric phase is not observable even with high cooling rates. For (b) AUUQU-5-N, a slight enthalpy change can be observed at around 35 °C. A transition into the SmZ_A phase is also observed for (c) AUUQU-7-N starting at around 27 °C. All of the transitions from the ordinary nematic to the antiferroelectric nematic phase are associated with only a miniscule change in enthalpy and are of monotropic nature.

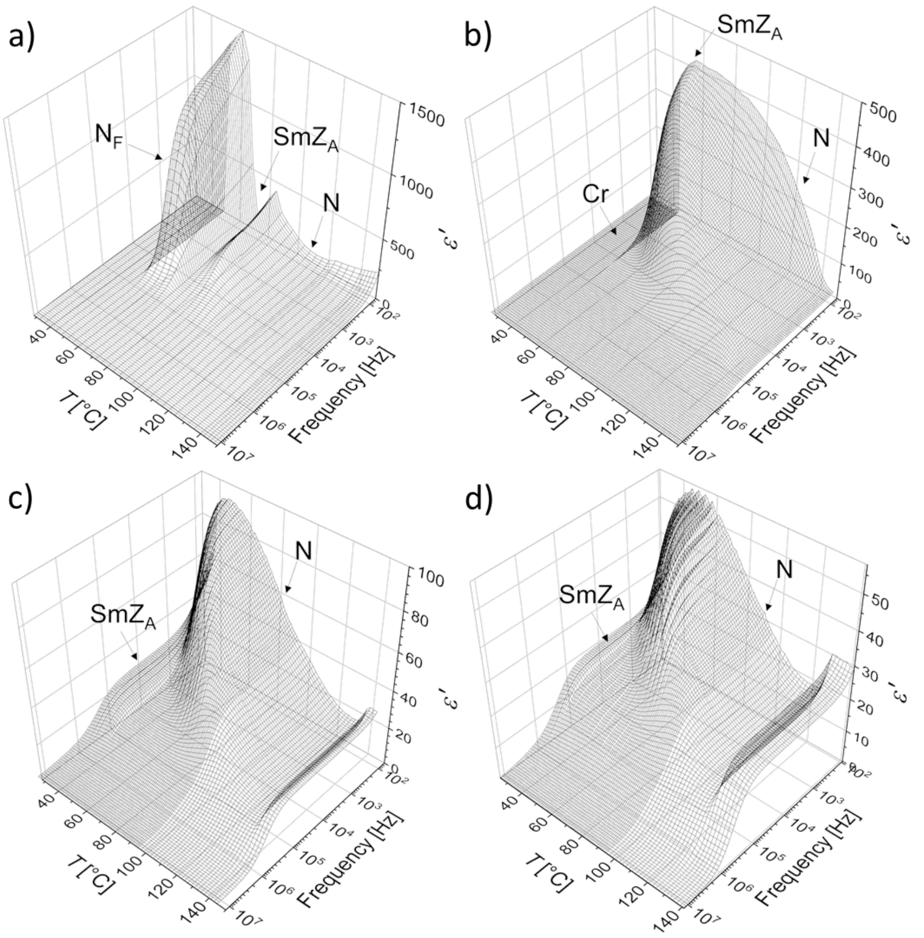

Figure S3: Measurements of the dielectric susceptibility ε' of AUUQU-*n*-N (*n* = 2, 3, 5, 7; (a) – (d)) measured in 20 µm thick cells with polyimide and ITO coating. All measurements were done while cooling. For (a) AUUQU-2-N, there is an increase in ε' just before the transition into the SmZ_A phase, with a narrow notch separating the ferroelectric from the ordinary nematic phase. For the other homologues, ε' also drops rapidly once the antiferroelectric phase is reached, showing similar behavior to AUUQU-2-N.

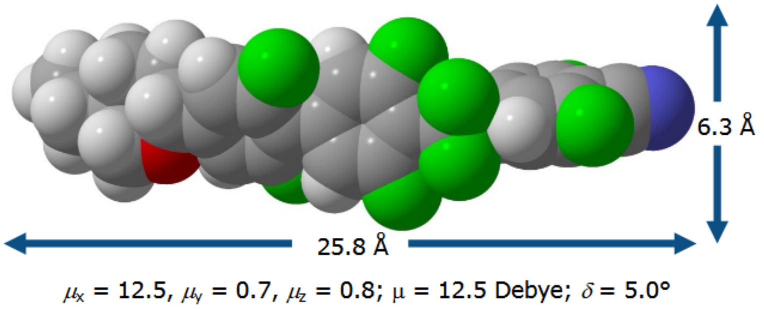

Figure S4: DFT calculations (m06-2X/6-31G(d)) for AUUQU-2-N showing the high longitudinal dipole moment of μ = 12.5 D.

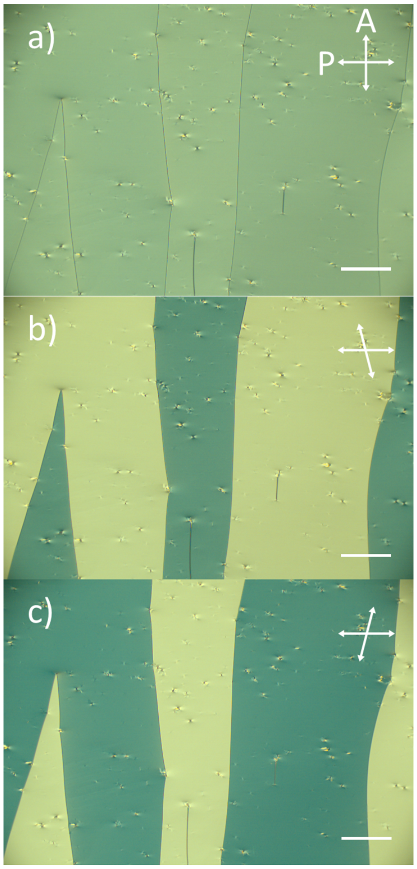

Figure S5: Optical textures of AUUQU-2-N in the N_F phase in 5 µm thick cells coated with polyimide and rubbed antiparallel on the two surfaces for planar alignment (all scale bars are 200 µm). In the N_F phase, millimeter-sized chiral domains are formed (a), the twist sense of which can be determined by decrossing the polarizers ((b) and (c)).

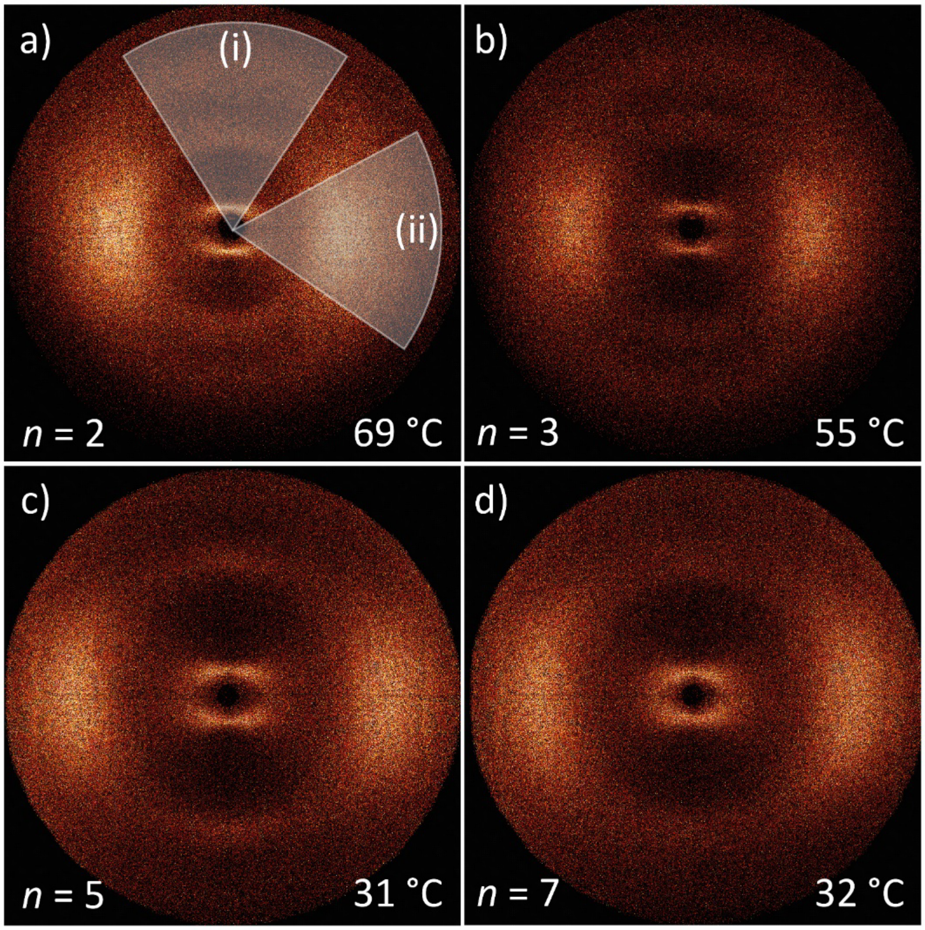

Figure S6: Wide-angle 2D X-ray patterns of selected homologs in the AUUQU-*n*-N series (n = 2, 3, 5, 7, (a) – (d)) obtained with a Bruker AXS NanoSTAR system. The images were taken in (a) the N_F and (b) – (d) the SmZ_A phase. The patterns are similar, but the small-angle signal is split to different extents. The grey cones represent different sector averages in χ that focus on either the additional signals along the meridian (i) or the equatorial scattering arc (ii).

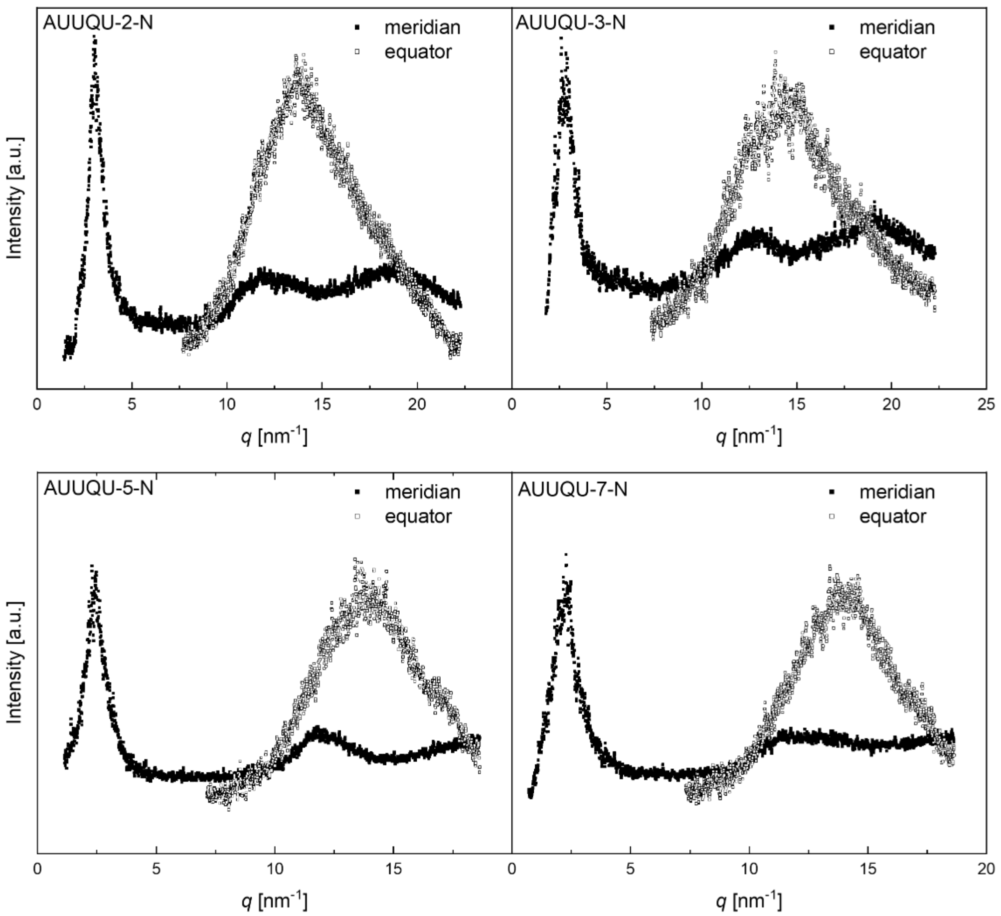

Figure S7: Sector averages with limited integration over χ of the X-ray data in Figure S6 for the homologous series AUUQU-*n*-N (n = 2, 3, 5, 7). The integrations were carried out on the meridian and on the equator, to distinguish the side-to-side and end-to-end stacking of the mesogens.

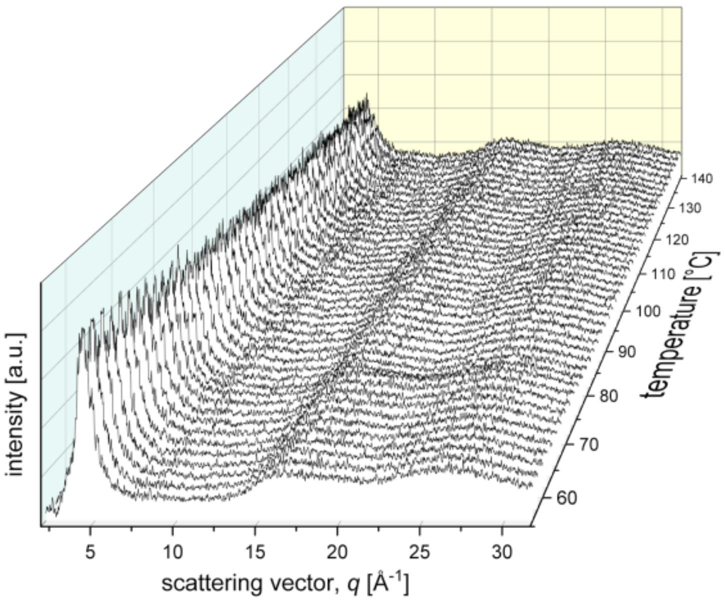

Figure S8: Sector averages (i) with limited integration over χ of the X-ray data in Figure S6 for AUUQU-2-N over the full temperature range.

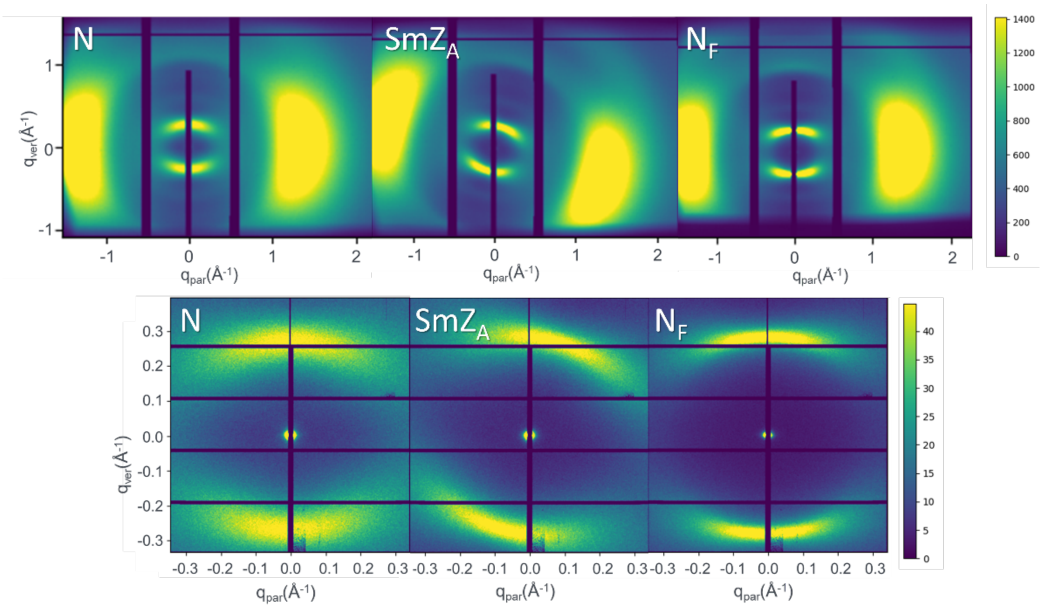

Figure S9: Synchrotron-based X-ray scattering of AUUQU-2-N in the N, SmZ_A and N_F phases obtained on the SMI microbeam line at NSLS II. The upper row depicts a broader *q* range. A rotation of the diffraction pattern can be observed in the SmZ_A phase.
